# Parkinsonism Following SARS‐CoV‐2 Infection Unmasks a Genetic Twist

**DOI:** 10.1002/mdc3.13785

**Published:** 2023-06-12

**Authors:** Simeona Jacinto, Anna Latorre, Francesca Magrinelli, Kailash P. Bhatia

**Affiliations:** ^1^ National Hospital for Neurology and Neurosurgery University College London Hospitals NHS Foundation Trust London United Kingdom; ^2^ Department of Clinical and Movement Neurosciences UCL Queen Square Institute of Neurology, University College London London United Kingdom

**Keywords:** COVID‐19, CADASIL, leukoencephalopathy, *NOTCH3*, parkinsonism

A broad spectrum of neurological sequelae has been associated with SARS‐CoV‐2 infection. Movement disorders remain uncommon, with higher prevalence of myoclonus, ataxia, and tremor.^1^ However, the observation of newly‐diagnosed parkinsonism following SARS‐CoV‐2 infection has stimulated debate whether this corroborates causality versus coincidence.^2^ We present a case with SARS‐CoV‐2 infection unmasking parkinsonism in the context of a previously undiagnosed monogenic disorder, which suggests that post‐SARS‐CoV‐2 parkinsonism should prompt clinicians to investigate alternative etiologies.

## Case Report

**Figure 1:**
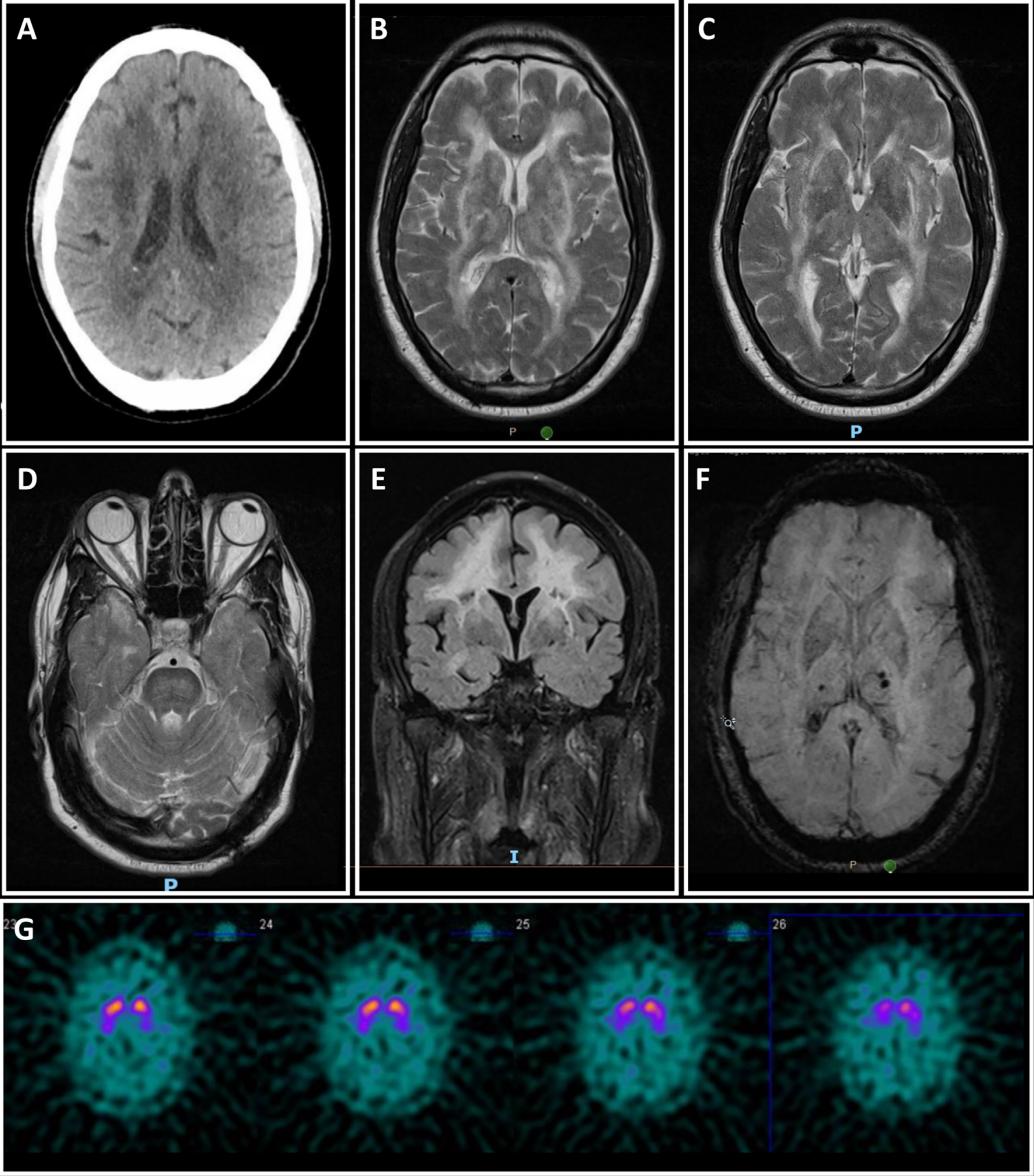
Serial neuroimaging. (**A**) Axial CT showing extensive symmetric white matter hypodensity. (**B–D**) Axial T2 MRI shows extensive symmetric white matter hyperintensity more pronounced in the frontal and parietal lobes, with periventricular and subcortical involvement, as well as patchy hyperintensity in the basal ganglia, external capsule, anterior temporal poles, and pons. (**E**) Coronal T2 MRI showing extensive symmetric white matter hyperintensity and patchy hyperintensity in the right temporal lobe. (**F**) Axial SWI MRI shows few microhaemorrhages within bilateral thalami, with further scatter microhaemorrhages. (**G**) DaTscan reveals reduced dopaminergic uptake bilaterally that is more prominent in left putamen.

A 64‐year‐old male presented with fever, cough, dyspnoea, lethargy and confusion which developed over one week. SARS‐CoV‐2 pneumonitis was diagnosed, and rapid deterioration required mechanical ventilation. Even after sedation weaning, his level of consciousness remained attenuated (GCS 6/15 with tracheostomy in‐situ). Brain CT demonstrated extensive symmetric white matter disease (Fig. 1A). EEG suggested diffuse cerebral dysfunction/encephalopathy.

Clinical status remained poor with severe global cognitive impairment, hypophonia (partial left vocal cord palsy related to intubation), aphasia, dysphagia, quadriparesis (worse on the right), and double incontinence. After three months, parkinsonism developed with hypomimia, bradykinesia, rigidity, shuffling gait and freezing. Pseudobulbar affect was observed. Serial brain MRIs showed stable symmetric and extensive T2/FLAIR white matter hyperintensity in the frontal and parietal lobes. Patchy signal abnormalities were seen in the temporal and occipital lobes, capsular regions, basal ganglia and thalami, pons, cerebral peduncles, and middle cerebellar peduncles, with lacunes being evident (Fig. 1B–E). MRI‐SWI demonstrated microhaemorrhages (Fig. 1F). A DaTscan revealed reduced dopaminergic uptake bilaterally, worse on the left (Fig. 1G). Blood/CSF investigations (Table S1), NCS/EMG and autonomic testing were unremarkable. Neurocognitive assessment showed global cognitive impairment, mostly in anterior and subcortical regions.

Levodopa (100 mg TDS) improved his mobility significantly, while cognition and communication remained considerably affected and severe hypophonia persisted despite vocal cord palsy resolution. After one year, he experienced an acute episode of confusion and anarthria followed by general regression. Brain MRI with DWI (5 days post episode) remained unchanged. After increasing levodopa to 500 mg/day, he gradually recovered to his preceding deficient baseline.

Past medical history included hypercholesterolaemia and previous Miller‐Fisher syndrome. He had no prodromal parkinsonian features, migraines, seizures, significant cognitive dysfunction, or neurotropic medication use. His father died after recurrent strokes in his 50s.

Neurological examination (Video 1) revealed hypomimia, pseudobulbar affect (inappropriate laughing) and barely audible whispered one‐word responses. He had gaze impersistence. No tremor, dystonia, or dysmetria was found. He had asymmetric (worse on right) bradykinesia and rigidity. His power was normal with globally brisk reflexes, flexor plantar responses, and absent clonus. There were no sensory abnormalities. His postural reflexes were impaired. He had a shuffling gait with slightly stooped posture and bilaterally reduced arm swing.

**Video 1:** Neurological examination revealed hypomimia and gaze impersistence. There was no tremor, dystonia or dysmetria. He had asymmetrical bradykinesia, worse on the right. His power was normal, with globally brisk reflexes, flexor plantar responses and no clonus. No sensory abnormalities were found. He had a shuffling gait with slightly stooped posture and bilaterally reduced arm swing. There was motor improvement after 1 year on benzeraside/levodopa 25/100 mg one and a half tablets twice a day during the daytime and two tablets once at bedtime, which was likely also attributable to recovery from encephalopathy and rehabilitation.

His clinical presentation was initially attributed to hypoxic ischemic damage and/or SARS‐CoV‐2‐related leukoencephalopathy. Diffuse small vessel disease with either monogenic or sporadic etiology was proposed given his stable serial neuroimaging findings. The former was considered more likely given his suggestive family history and minimal vascular risk factors. Genetic testing detected a heterozygous pathogenic variant NM_000435.3:c.1364G > T p.(Cys455Phe) in *NOTCH3*, which is associated with cerebral autosomal dominant arteriopathy with subcortical infarcts and leukoencephalopathy (CADASIL).

## Discussion

This case uniquely illustrates post‐SARS‐CoV‐2 parkinsonism heralding previously undiagnosed CADASIL. Severe SARS‐CoV‐2‐related pneumonitis and encephalopathy preceded a prolonged clinical course with severe cognitive and motor deficits and evolution to asymmetric parkinsonism with evidence of presynaptic nigrostriatal dysfunction. Key features prompting further investigation for monogenic vasculopathy included neuroimaging suggestive of extensive small vessel disease, history of early recurrent strokes in a first‐degree relative, and absence of significant vascular risk factors.

CADASIL is a rare autosomal dominant disorder due to loss‐of‐function *NOTCH3* variants leading to accumulation of abnormal transmembrane deposits on vascular smooth muscle cells and impaired vascular function. Its usual stepwise progression includes migraine with aura, transient ischemic attacks, ischemic strokes, and dementia.^3^, ^4^ Parkinsonism is a late albeit not rare manifestation^5^ characterized by progressive supranuclear palsy‐like syndrome, lower body and asymmetric motor involvement, cognitive impairment and limited levodopa response.^6^ DaTscan can demonstrate reduction of tracer uptake in the putamen with inconstant caudate involvement.^5^ Although most cases of parkinsonism in CADASIL were not levodopa responsive, there is little evidence of limited levodopa response in very few reports. In this patient, diffuse small vessel disease involving the nigrostriatal or thalamocortical pathways (Fig. 1) might have caused presynaptic dopaminergic, as documented by his abnormal DaTscan, and explain a favorable response to levodopa.

A literature review identified a total of 20 cases of post‐SARS‐CoV‐2 parkinsonism, but CADASIL was never diagnosed.^7^ We identified six cases of CADASIL decompensation due to SARS‐CoV‐2 infection, and parkinsonism was not reported in any of these (Table 1). It is unclear whether these is the same series reported by Noui et al.^8^

**TABLE 1:** Cases of CADASIL Presentations or Deterioration Associated with SARS‐CoV‐2 Infection

Background	Neurological presentation, PMH and FH	CADASIL diagnosis	Reference
F, 30	Headache, dysarthria, psychomotor slowness. PMH: Spastic right hemiparesis and paresis of right abducens nerve (deficit from two years old, never radiologically diagnosed).	New diagnosis. *Heterogeneous missense variant NOTCH3 (NM_000435.3) c.1320C > G (p.Cys440Trp) in exon 8 (NC_000019.10:g.15189047G > C)*.	_3_
F, 37	Dysarthria, left lower limb weakness, ataxia. Right pontine infarct. PMH: CADASIL, hypertension, migraines, smoking.	Previously stable genetically proven CADASIL. *Heterozygous missense mutation NOTCH3 c.3062A > G; p.Tyr1021Cys*.	^4^
F, 38	Dysarthria, right facial droop, dysphagia, right hemiparesis. Multiple acute bilateral border zone infarcts. FH: CADASIL (father and sister).	New diagnosis. *Heterogeneous pathogenic missense variant NOTCH3 c.268C > T (p.Arg90Cys)*.	^8^
F, 45	Presentation: Dysarthria, confusion. Multiple acute internal border zone infarcts FH: father had stroke at 52 years old.	New diagnosis. *Heterogeneous pathogenic missense variant NOTCH3 confirmed*.	^9^
F, early 40s	Presentation: Headache, dysphagia, dysarthria, encephalopathy. Acute multi‐infarct encephalopathy.	New diagnosis. *Heterogeneous pathogenic missense variant NOTCH3 confirmed*.	^10^
F, 60	Presentation: Aphasia, agraphia, worsened right upper limb weakness. Left corona radiata ischaemic stroke. PMH: CADASIL (stable right sided weakness from 4 years prior), hypertension.	Previously stable genetically proven CADASIL. *Heterozygous missense mutation NOTCH3 c.994C > T (p.Arg332Cys)*.	^11^

Abbreviations: CADASIL, cerebral autosomal dominant arteriopathy with subcortical infarcts and leukoencephalopathy; F, female; FH, family history; NOTCH3, neurogenic locus notch homolog protein 3; PMH, past medical history; SARS‐CoV‐2, severe acute respiratory syndrome coronavirus 2.

This is the first description of post‐SARS‐CoV‐2 levodopa‐responsive parkinsonism in the context of CADASIL. We believe that parkinsonism is attributable to CADASIL, that was unmasked by SARS‐CoV‐2 infection. Extensive CADASIL small vessel arteriopathy can affect the structural and functional integrity of the basal ganglia thereby causing vascular parkinsonism. SARS‐CoV‐2 infection could trigger this process by creating an inflammatory environment predisposing vascular endothelial injury.^9^

This case highlights the importance of investigation for other etiologies in cases of post‐SARS‐CoV‐2 parkinsonism and supports the hypothesis that SARS‐CoV‐2 infection can unmask parkinsonism from other aetiologies, rather than directly causing it.

## Author Roles

(1) Research project: A. Conception, B. Organization, C. Execution; (2) Data Analysis: A. Design, B. Execution, C. Review and Critique; (3) Manuscript Preparation: A. Writing of the first draft, B. Review and Critique.

S.J.: 1C, 3A.

A.L.: 1C, 3B.

F.M.: 1C, 3B.

K.B.: 3B.

## Disclosures

**Ethical Compliance Statement:** We confirm that we have read the Journal's position on issues involved in ethical publication and affirm that this work is consistent with those guidelines. The authors confirm that the approval of an institutional review board was not required for this work. We confirm that the patient and his family provided consent for video acquisition and publication.

**Funding Sources and Conflicts of Interest:** The authors declare that there are no conflicts of interest relevant to this work.

**Financial Disclosures for the Previous 12 Months:** Simeona Jacinto and Anna Latorre have no financial disclosures. Francesca Magrinelli is supported by the Edmond J. Safra Foundation. Kailash P. Bhatia has received grant support from Welcome/MRC, NIHR, Parkinson's UK and EU Horizon 2020. He receives royalties from publication of the Oxford Specialist Handbook Parkinson's Disease and Other Movement Disorders (Oxford University Press, 2008), of Marsden's Book of Movement Disorders (Oxford University Press, 2012), and of Case Studies in Movement Disorders–Common and uncommon presentations (Cambridge University Press, 2017). He has received honoraria/personal compensation for participating as consultant/scientific board member from Ipsen, Allergan, Merz and honoraria for speaking at meetings and from Allergan, Ipsen, Merz, Sun Pharma, Teva, UCB Pharmaceuticals and from the American Academy of Neurology and the International Parkinson's Disease and Movement Disorders Society.

## Supporting information


**Table S1.** Blood and CSF results. ANA, antinuclear antibodies; ANCA, antineutrophil cytoplasmic antibodies; Caspr2, contactin‐associated protein‐like 2; CMV, cytomegalovirus; CSF, cerebrospinal fluid; DPPX, dipeptidyl‐peptidase‐like protein 6; dsDNA, double stranded DNA; EBV, Epstein–Barr virus; GAD, glutamic acid decarboxylase; HBV, hepatitis B virus; HCV, hepatitis C virus; HIV, human immunodeficiency virus; HSV, herpes simplex virus; IgG, immunoglobulin G; IgLON5, immunoglobulin‐like cell adhesion molecule 5; LGI1, leucine‐rich glioma‐inactivated 1; NMDA, N‐methyl‐D‐aspartate; JCV, John Cunningham virus; LDH, lactate dehydrogenase; OCB, oligoclonal bands; P‐tau, phosphorylated tau; VZV, varicella‐zoster virus; WBC, white blood cells. * Underlined values are abnormal.
