# Supplementary material for: Parkinsonism Following SARS‐CoV‐2 Infection Unmasks a Genetic Twist
Source: Mov Disord Clin Pract. 2023 Jun 12;10(7):1146–9. doi: 10.1002/mdc3.13785 (PMC10354609; doi:10.1002/mdc3.13785)
Supplement: Supplementary file 1 — Table S1. Blood and CSF results. ANA, antinuclear antibodies; ANCA, antineutrophil cytoplasmic antibodies; Caspr2, contactin‐associated protein‐like 2; CMV, cytomegalovirus; CSF, cerebrospinal fluid; DPPX, dipeptidyl‐peptidase‐like protein 6; dsDNA, double stranded DNA; EBV, Epstein–Barr virus; GAD, glutamic acid decarboxylase; HBV, hepatitis B virus; HCV, hepatitis C virus; HIV, human immunodeficiency virus; HSV, herpes simplex virus; IgG, immunoglobulin G; IgLON5, immunoglobulin‐like cell adhesion molecule 5; LGI1, leucine‐rich glioma‐inactivated 1; NMDA, N‐methyl‐D‐aspartate; JCV, John Cunningham virus; LDH, lactate dehydrogenase; OCB, oligoclonal bands; P‐tau, phosphorylated tau; VZV, varicella‐zoster virus; WBC, white blood cells. * Underlined values are abnormal. [file MDC3-10-1146-s001.docx]

**Supplementary Table: Blood and CSF results**

| **Blood results** | | | | | |
| --- | --- | --- | --- | --- | --- |
| **HIV, HBV, HCV, CMV, EBV, HSV, VZV, JCV, Syphilis** | Negative | **Anti-NMDA receptor, -GAD, Glycine, -DPPX, -LGI1, -IgLON5, -Caspr2, paraneoplastic antibodies (Hu, Ri, Tr, Yo)** | | | Negative |
| **Long chain fatty acids** | Normal | **ANCA** | | | Negative |
| **Amino acid profile** | Normal | **ANA*** | | | Positive (1:80 hom) |
| **White cell enzymes** | Normal | **Anti-dsDNA IgG** | | | <0.6 (0 – 10 IU/ml) |
| **Urine bile acid** | Negative |  | | |  |
| **CSF results** | | | | | |
| **WBC** | 2 (0 - 5 cells/uL) | **Gram stain** | Nil | **Neurofilament** | 5, 63 (0 - 1,782 pg/mL) |
| **Protein** | 0.59 (0.13 -0.45 g/L) | **CSF culture** | Negative | **Tau/P-tau ratio** | 8.78 |
| **Glucose** | 3.31 (2.2 - 4.2 mmol/L) | **HSV, VZV, CMV, JCV** | Negative | **Beta amyloid ratio 1-42/1-40** | 0.046 |
| **Cytology** | Nil | **LDH** | 43 (0 - 57 U/L) | **Protein 14-3-3** | Negative |
| **OCB** | Negative | **Lactate** | 1.38 (1.1 - 2.4 mmol/L) |  |  |
| **IgG Index** | 0.47 (10 - 40 mg/L) | **Anti-GAD, -DPPX, -NMDA receptor antibodies** | Negative |  |  |

Legend: ANA = antinuclear antibodies; ANCA = antineutrophil cytoplasmic antibodies; Caspr2 = contactin-associated protein-like 2; CMV = cytomegalovirus; CSF = cerebrospinal fluid; DPPX = dipeptidyl-peptidase-like protein 6; dsDNA = double stranded DNA; EBV = Epstein-Barr virus; GAD = glutamic acid decarboxylase; HBV = hepatitis B virus; HCV = hepatitis C virus; HIV = human immunodeficiency virus; HSV = herpes simplex virus; IgG = immunoglobulin G; IgLON5 = immunoglobulin-like cell adhesion molecule 5; LGI1 = leucine-rich glioma-inactivated 1; NMDA = N-methyl-D-aspartate; JCV = John Cunningham virus; LDH = lactate dehydrogenase; OCB = oligoclonal bands; P-tau = phosphorylated tau; VZV = varicella-zoster virus; WBC = white blood cells. * Underlined values are abnormal.
